# The Relationship Between Nursing Students' Microplastic Pollution Awareness and Environmental Literacy: A Cross‐Sectional Study

**DOI:** 10.1002/nop2.70735

**Published:** 2026-08-10

**Authors:** Fatma Ceylan Çiray, Ayşe Altınkaynak, Fatma Türedi, Tülay Erciyes

**Affiliations:** ^1^ Department of Public Health Nursing, Faculty of Nursing Hacettepe University Ankara Turkey; ^2^ Faculty of Nursing Hacettepe University Ankara Turkey

**Keywords:** environmental awareness, environmental health, environmental literacy, microplastics, nursing students

## Abstract

**Aim:**

This study aims to examine the relationship between microplastic pollution awareness and environmental literacy levels among undergraduate nursing students.

**Design:**

The study was conducted using a cross‐sectional design.

**Methods:**

This study included 356 undergraduate nursing students selected from each academic year using proportional stratified sampling. Data were collected between March and June 2023 using a demographic form, the Microplastic Pollution Awareness Scale, and the Environmental Literacy Scale. Data analysis was performed using SPSS 23.0, with descriptive statistics, Mann–Whitney *U*, Kruskal–Wallis, and Spearman correlation analyses.

**Results:**

The mean microplastic pollution awareness score and environmental literacy score were 22.98 ± 4.85 and 85.21 ± 10.35, respectively. Participants who expressed willingness to receive environmental education and those who had previously heard of microplastics demonstrated higher mean scores for both microplastic pollution awareness and environmental literacy. A considerable proportion of participants reported not having previously heard of microplastics. Despite this, most nursing students (94.4%) exhibited high levels of environmental literacy. A positive correlation was found between microplastic pollution awareness and environmental literacy.

**Conclusion:**

This study highlights the potential importance of environmental literacy in raising nursing students' awareness of microplastic pollution. The current curriculum has limited content on environmental health, and it is mostly covered in a short section of the public health nursing course. These findings suggest that integrating content on environmental literacy and microplastic pollution across all stages of the nursing curriculum, as well as in in‐service training, may be beneficial.

**Implications for Nursing Practice:**

Strengthening environmental literacy in nursing education may improve awareness of microplastic pollution and support nurses in promoting environmentally responsible behaviours and environmental health in clinical and community settings.

**Impact:**

This study addresses the limited evidence on microplastic pollution awareness among nursing students. The findings highlight the importance of environmental literacy in shaping awareness and can inform curriculum development and educational strategies targeting future nurses.

**Reporting Method:**

This study was reported in accordance with the STROBE guidelines.

**Patient or Public Contribution:**

No patient or public involvement.

## 1 Introduction

With the growth of the world population and developments in technology and industry, the amount of waste left to nature is increasing every year. Recently, the amount of waste from plastic products, which have increased in use due to their lightweight, affordability, durability, and ease of transport, has reached an alarming level (Kibria et al. 2023). The use of plastics has continuously increased from the 1950s to the present day, resulting in environmental pollution and waste problems (Worm et al. 2017). Plastics thrown into the environment do not only create visual pollution; these products also pose a risk to the environment and living things by breaking down into smaller sizes called ‘microplastics’ (Yee et al. 2021). Microplastics (MP) are plastic particles less than 5 mm in size (Arthur et al. 2009). Recent studies show that microplastics are found primarily in aquatic ecosystems (Bertoldi et al. 2021; Li et al. 2021), in soil (Ding et al. 2021), in the air and atmosphere (Huang et al. 2020; Szewc et al. 2021). Today, microplastics can even be found in glaciers (Bergmann et al. 2019). Microplastics have become a serious global problem due to their ubiquity in nature and persistence in the environment (Bertoldi et al. 2021).

Microplastics are inherently toxic to organisms and, therefore, negatively affect human, animal, and environmental health (Laskar and Kumar 2019; Yee et al. 2021). Humans are exposed to many types of microplastics through inhalation, digestion, and skin contact (Lei et al. 2024; Yee et al. 2021). Recent studies have shown that microplastics have been detected in vital human tissues such as blood, the lung, the heart, and even in human faeces (Lei et al. 2024; Schwabl et al. 2019). Studies have identified serious problems such as respiratory and intestinal infections, immune system disorders, neurological disorders, and inflammation in humans exposed to microplastics (Bhuyan 2022; Gasperi et al. 2018; Prata et al. 2020; Yee et al. 2021). Chronic exposure to MPs causes deaths and various health problems in susceptible individuals even at low doses (Prata 2018; Prata et al. 2020).

In response to the increasing threat of microplastic pollution, there is a pressing need for new strategies to protect human, animal, and environmental health. One promising approach is the promotion of environmental literacy (EL). Environmental literacy, defined for the first time by Charles E. Roth in 1968 (Roth 1992) refers to an individual's knowledge, sensitivity, skills, and behaviours about the environment in which the individual is located. In line with this definition, environmental literacy plays an important role in raising environmental awareness in individuals, learning the laws of nature, and protecting and improving the health of ecosystems (Kapan and Gürel 2022). People with higher levels of environmental literacy are more sensitive, more conscious, and more aware of the environment (Örs 2022). In this context, environmental literacy can contribute to a better understanding of emerging environmental issues, such as microplastic pollution, by enabling individuals to access, evaluate, and interpret environmental information, thereby raising awareness about the sources, pathways of spread, and potential health effects of these issues.

The environmental literacy levels of nurses, who constitute the largest workforce in the health sector, are important because they play an active role in both individual and social environmental health education (Cengiz and Bahar 2023). Nurses and nursing students occupy a critical role in environmental health through their responsibilities in health promotion, risk awareness, and preventive practices related to climate change, indoor environmental hazards, and microplastic pollution (Amin et al. 2024; Ediz and Uzun 2025; Incesu and Yas 2024; Pratesi et al. 2026). Enhancing environmental literacy in nursing begins with educating undergraduate nursing students, who will become future healthcare professionals (McElroy et al. 2021). Nursing students with high environmental literacy will be better prepared for the environmental problems they will encounter in their professional lives (Örs 2022). Studies examining the environmental literacy levels of nursing students are available in the literature. Studies show that nursing students have high (Amin et al. 2024; Cengiz and Bahar 2023; Dağal and Fırat Kılıç 2023; Incesu and Yas 2024; Yusuf et al. 2022) and medium (Gök and Kiliç 2021; Kapan and Gürel 2022) levels of environmental literacy.

However, studies conducted with nursing students on plastics and plastic pollution have shown that nursing students lack sufficient awareness and knowledge (Ravi et al. 2025; Ravi, Edwin, and Jaison 2024; Ravi, Edwin, Rasheed, et al. 2024). Furthermore, it has been observed that these studies primarily address the issue through the concept of plastic and do not directly focus on awareness of microplastic pollution. Despite the growing recognition of microplastic pollution as an emerging public health threat, evidence regarding microplastic pollution awareness among nursing students remains limited. There is a significant gap in studies investigating awareness of microplastic pollution among nursing students, particularly in Turkey, making this study necessary. Given that nurses play a critical role in environmental health promotion and patient education, understanding their awareness of emerging environmental threats, such as microplastics is essential for effective nursing practice.

Moreover, to the best of our knowledge, no study has examined the relationship between microplastic pollution awareness and environmental literacy in this population. This study contributes to the existing literature by addressing this gap and providing evidence to inform the integration of environmental and microplastic‐related content into nursing education. Therefore, this study aims to examine the relationship between microplastic pollution awareness and environmental literacy levels among undergraduate nursing students.

### 1.1 Research Questions

What are the microplastic pollution awareness levels among nursing students?

What are the environmental literacy levels among nursing students?

What is the relationship between microplastic pollution awareness and environmental literacy in nursing students?

## 2 Methods

### 2.1 Study Design

This study employed a cross‐sectional design. The STROBE guidelines were used in the reporting process.

### 2.2 Sample Size and Study Participants

This study was conducted at the Faculty of Nursing of a state university in Türkiye that offers a four‐year undergraduate nursing program.

The population of this study consisted of first‐, second‐, third‐, and fourth‐grade undergraduate nursing students enrolled in the program (*N* = 817). The minimum required sample size was calculated as 262 using the finite population sample size formula, with a significance level of *α* = 0.05 and a power of 95%.

The number of participants for each academic year was determined using a proportional stratified sampling method, and volunteer participants who met the inclusion criteria were enrolled in the study using a convenience sampling approach until the target sample size was reached. Since the concept of microplastics is relatively new, it was planned to include 30% more participants than the calculated sample size.

Because the main analyses of the study were based on correlational statistics, the adequacy of the final sample size was also evaluated for correlation analysis. A post hoc power analysis was conducted using G*Power 3.1 based on the observed correlation coefficient (*r* = 0.313), using a two‐tailed *α* level of 0.05 and a total sample size of 356. The achieved statistical power was 0.99, indicating that the sample size was sufficient to detect a moderate correlation. According to Cohen's criteria, this corresponds to a moderate effect size.

As a result, 86 students from 1st grade, 92 students from 2nd grade, 92 students from 3rd grade, and 86 students from 4th grade were included in the study. The study was completed with a total of 356 nursing students.

Inclusion criteria were as follows: (1) being an undergraduate nursing student enrolled in the faculty during the data collection period, (2) being aged 18 years or older, (3) being able to understand and respond to the questionnaire, and (4) providing voluntary informed consent to participate in the study.

Exclusion criteria included: (1) incomplete or missing questionnaire data, and (2) refusal to participate or withdrawal from the study at any stage.

### 2.3 Instruments

#### 2.3.1 Demographic Form

This form, developed by the researchers through a literature review, consists of nine questions in total. These questions assessed nursing students' demographic characteristics, whether they had heard of microplastics, membership in environmental organizations, and whether they had received environmental health education and were willing to accept such education.

#### 2.3.2 Microplastic Pollution Awareness Scale (MPAS)

The scale, developed by Güleşir and Gül 2022, is designed to assess awareness of microplastic pollution. The scale was used in its original language as developed by the authors and did not require any translation. The scale comprises 14 items distributed across three subscales: awareness of preventing microplastic pollution (5 items), awareness of the effects of microplastic pollution on organisms (5 items), and awareness of the effects of microplastic pollution on human health (4 items). The scale includes five negative and nine positive items. The Likert scale consists of 0 points = ‘no,’ 1 point = ‘not sure’, and 2 points = ‘yes’ options. The maximum score that can be obtained from the scale is 28. The higher the score, the higher the respondent's awareness of microplastic pollution. The Cronbach's *α* coefficient for the subscales is 0.74, 0.68, and 0.65, respectively (Güleşir and Gül 2022). This study's reliability coefficients were 0.810, 0.811, and 0.734 for the subscales, respectively, and 0.904 for the overall scale.

#### 2.3.3 The Environmental Literacy Scale for Adults (ELSA)

The scale developed by Atabek‐Yigit et al. in 2014 is designed to assess environmental literacy in adults. The scale comprises 20 items distributed across three subscales: environmental consciousness level (9 items), environmental anxiety level (6 items), and environmental awareness level (5 items). Each item is rated on a five‐point Likert scale, ranging from ‘strongly disagree’ (1) to ‘strongly agree’ (5), with two items reverse‐coded. The total score ranges from 20 to 100. Scores of 20–46 refers to low, 47–73 to medium, and 74–100 to high levels of environmental literacy. The categorization of environmental literacy levels (low, medium, and high) is based on the cut‐off points defined in the original scale development study by Atabek‐Yiğit et al. (2014). Exploratory factor analysis (EFA) revealed that the scale explained 47.17% of the variance. The Cronbach's α coefficient for the subscales is 0.807, 0.765, and 0.715, respectively, with an overall scale reliability of 0.881 (Atabek‐Yiğit et al. 2014).

This study's reliability coefficients were 0.859, 0.734, and 0.692 for the subscales, respectively, and 0.897 for the overall scale.

### 2.4 Data Collection

Data collection was conducted from the beginning of March until the end of June 2023.

During this period, educational activities across the country were conducted in a hybrid format due to the earthquakes that occurred in the Kahramanmaraş province of Türkiye. The sample size was calculated using proportional stratified sampling to ensure representation from each academic year (grades 1–4). For online data collection, students were contacted via class WhatsApp groups, informed about the study, and provided with a link (Google Survey) to access the questionnaire. Students who gave informed consent voluntarily completed the online questionnaire. The study was announced to students participating in educational activities at the school, and data were collected face to face (paper‐based) from volunteer students using questionnaire forms at appropriate times. Data collection continued until the predetermined sample size for each academic grade was reached. A total of 356 students participated in the study, corresponding to an approximate participation rate of 43.6%. Among the participants, 123 completed the questionnaire online, while 233 participated through face‐to‐face data collection. Given the use of voluntary participation and online recruitment via class communication groups, the exact number of students who received the survey invitation could not be determined.

Data were collected using the ‘Demographic Form’, ‘Microplastic Pollution Awareness Scale’ and ‘The Environmental Literacy Scale for Adults’. On average, participants dedicated about 10–15 min to complete the questionnaire.

### 2.5 Data Analysis

The statistical analysis of the data was performed using IBM SPSS 23.0. Categorical variables were evaluated using numbers and percentages, while descriptive statistics (mean and standard deviation) were used for numerical variables with a normal distribution. For numerical variables that did not follow a normal distribution, the median and interquartile range (IQR) were applied. Compliance with normal distribution was evaluated using the Kolmogorov–Smirnov test and histogram plots method, and it was seen that the data were not normally distributed. Spearman correlation analysis was performed to evaluate relationships between variables. The Mann–Whitney *U* and Kruskal–Wallis tests were utilized to analyze the correlation between categorical variables and scale scores. The analyses presented in Table 1 were considered exploratory; therefore, no adjustment for multiple comparisons (e.g., Bonferroni correction) was applied. Significance was accepted as 0.05 in statistical analyses.

**TABLE 1:** Socio‐demographic characteristics of participants and differences in mean microplastic pollution awareness and environmental literacy scores.

Variable			Microplastic pollution awareness		Environmental literacy	
	*N*	%	Mean ± SD	Median (IQR)	Mean ± SD	Median (IQR)
Gender						
Female	297	83.4%	23.35 ± 4.67	25 (21–27)	85.80 ± 9.83	88 (81–92)
Male	59	16.6%	21.14 ± 5.32	22 (15–26)	82.27 ± 12.33	85 (77–91)
*Z (p)*			**−3.07 (0.002)**		−1.96 (0.050)	
Marital status						
Married	0	—	—		—	
Not married	356	100%	22.98 ± 4.85	25 (20–27)	85.21 ± 10.35	87 (80–92)
Academic year						
1st year	86	24.2%	23.42 ± 4.34	25 (22–27)	82.49 ± 12.51	84.5 (78–91)
2nd year	92	25.8%	23.15 ± 4.54	25 (21–27)	85.48 ± 10.59	88 (81–92)
3rd year	92	25.8%	22.35 ± 5.36	25 (18–27)	86.70 ± 7.34	87 (81–92.50)
4th year	86	24.2%	23.05 ± 5.09	25 (21–27)	86.07 ± 10.11	88.5 (82–92)
*X* ^*2*^ *(p)*			0.999 (0.802)		7.57 (0.056)	
Member of an environmental organization						
Yes	18	5.1%	24.61 ± 3.05	25 (23–27)	90.72 ± 5.99	90.5 (88–97)
No	338	94.9%	22.90 ± 4.91	25 (20–27)	84.92 ± 10.45	87 (80–91)
*Z (p)*			−1.166 (0.244)		−2.73 (**0.006**)	
If yes, name of environmental organization						
AFAD	1	5.55%				
Greenpeace	1	5.55%				
TEMA	16	88.88%				
Receiving course on environmental health and problems						
Yes	29	8.1%	22.45 ± 5.27	25 (16–27)	87.28 ± 8.17	89 (85–92)
No	327	91.9%	23.03 ± 4.82	25 (20–27)	85.03 ± 10.51	87 (80–92)
*Z (p)*			−0.508 (0.611)		−1.19 (0.233)	
If yes, the name of the received course						
Environmental health	24	82.75%				
First aid	1	3.44%				
Occupational health	1	3.44%				
Health promotion	1	3.44%				
Improving public health	2	6.89%				
Willingness to receive environmental health education						
Yes	237	66.6%	23.49 ± 4.45	25 (22–27)	86.33 ± 9.88	88 (82–92)
No	119	33.4%	21.98 ± 5.45	24 (17–27)	82.99 ± 10.94	84 (78–90)
*Z (p)*			**−2.214 (0.027)**		**−3.60 (0.000)**	
Hearing the concept of microplastic						
Yes	113	31.7%	25.42 ± 3.39	26 (25–28)	87.28 ± 10.97	89 (85–94)
No	243	68.3%	21.85 ± 5.01	23 (17–26)	84.25 ± 9.92	85 (79–91)
*Z (p)*			**−7.074 (0.000)**		**−3.91 (0.000)**	
If the status of hearing the concept of microplastics is ‘yes’, the place of hearing the concept *						
Research/articles	13	10.74%				
Documentary	6	4.95%				
I don't know/don't remember	15	12.40%				
Environmental health course	2	1.65%				
News	7	5.78%				
Internet	18	14.87%				
High School	6	4.95%				
School/course	15	12.40%				
Social Media	34	28%				
Civil society organization	1	0.8%				
Television	4	3.3%				

*Note:* Z, Mann–Whitney *U*; a. KruskalWallis Test; *p* < 0.05 statistical significance.

Abbreviations: IQR, interquartile range; *X^2^*, Chi‐Square.

* Students gave more than one response.

### 2.6 Ethical Considerations

The study received approval from the Research Ethics Commission (E‐35853172‐300‐00002516592) on 17 December 2022. In addition, institutional permission was obtained from the Faculty of Nursing, and permission was also obtained from the owners of the scales to use the scales in the study. The research adhered to the ethical guidelines of the Declaration of Helsinki. All participants were thoroughly informed about the study's objectives and procedures, and they provided written informed consent before participating in the study. Special attention was given to protecting vulnerable participants, particularly those who might feel coerced into participating due to the power dynamics inherent in an academic setting.

## 3 Result

### 3.1 Socio‐Demographic Characteristics and Distribution of Microplastic Pollution Awareness and Environmental Literacy Scores

The mean age of the students participating in the study was 21.84 ± 1.53 years (min: 18, max: 28). Eighty‐three per cent of the participants were female, and 16.6% were male (Table 1).

The majority of participants (94.9%) reported that they were not members of any environmental organization. Additionally, 91.9% of the students stated that they had not taken any courses related to environmental health. Among those who had, most had taken a course titled *Environmental Health*. When asked whether they would like to receive training on environmental health, 66.6% of the participants responded affirmatively. Regarding the concept of microplastics, 68% of the students reported that they had never heard of the term before. Among those who had heard of the concept (28%), the most common source of information was social media platforms (Table 1).

While gender did not make a significant difference to the environmental literacy score, the mean score for microplastic pollution awareness was significantly higher in female students (*p* < 0.05). There were no statistically significant differences in environmental literacy or microplastic pollution awareness scores across different academic years or between students who had or had not received environmental health education. However, students who expressed a desire to receive environmental education and had previously heard of microplastics scored significantly higher on both scales (*p* < 0.05). Membership in an environmental organization was not significantly associated with microplastic pollution awareness scores, but was associated with significantly higher environmental literacy scores (*p* < 0.05). The mean age of the participants was 21.84 ± 1.53 years (range: 18–28).

### 3.2 Microplastic Pollution Awareness and Environmental Literacy Scores and Subscale Scores

The mean score of the participants' awareness of microplastic pollution was 22.98 ± 4.85 (range: 6–28). In addition, the mean score for awareness of level regarding the prevention of microplastic pollution, which are the sub‐dimensions of the scale, was 8.69 ± 1.79 (range: 2–10); the mean score of the awareness level regarding the effects of microplastic pollution on living things was 8.20 ± 1.96 (range: 0–10); and the mean score of the awareness level regarding the effects of microplastic pollution on human health was 6.10 ± 1.65 (range: 0–8) (Table 2).

**TABLE 2:** Microplastic pollution awareness and environmental literacy and subscale mean scores.

Study measures	Mean ± SD	Minimum	Maximum	Median (IQR)
MPAS (total)	22.98 ± 4.85	6.00	28.00	25 (20–27)
Awareness on preventing microplastic pollution	8.69 ± 1.79	2.00	10.00	9 (8–10)
Awareness of the effects of microplastic pollution on organisms	8.20 ± 1.96	0.00	10.00	9 (7–10)
Awareness on the effects of microplastic pollution on human health	6.10 ± 1.65	0.00	8.00	6 (5–7)
ELSA (total)	85.21 ± 10.35	20.00	100.00	87 (80–92)
Environmental consciousness	40.52 ± 5.11	9.00	45.00	42 (38–44)
Environmental anxiety	25.99 ± 3.40	6.00	30.00	26 (24–28)
Environmental awareness	18.70 ± 3.42	5.00	25.00	19 (17–21)

Abbreviation: SD, standard deviation.

The total mean environmental literacy score of all participants was 85.21 ± 10.35 (range: 20–100), indicating that nursing students had a high level of environmental literacy. In addition, the mean scores of the sub‐dimensions of the scale were found to be 40.52 ± 5.11 (range: 9–45) in the environmental consciousness sub‐dimension, 25.99 ± 3.40 (range: 6–30) in the environmental anxiety sub‐dimension, and 18.70 ± 3.42 (range: 5–25) in the environmental awareness sub‐dimension (Table 2).

**FIGURE 1:**
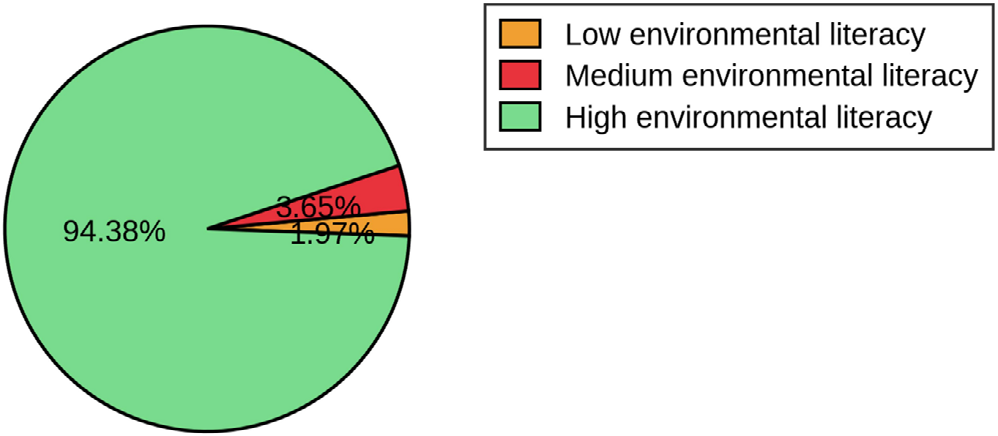
Categories according to mean scores of environmental literacy*. *Cut‐off values were adopted from Atabek‐Yiğit et al. (2014).

When participants were categorized based on their environmental literacy scores, 2% were classified as having low environmental literacy, 3.65% had medium environmental literacy, and 94.38% were classified as having high environmental literacy (Figure 1).

### 3.3 Evaluation of the Relationship Between Microplastic Pollution Awareness Level Sub‐Dimensions and Environmental Literacy Level Sub‐Dimensions

The results showed that there was a significant positive correlation between microplastic pollution awareness and environmental literacy (*r* = 0.313, *p* < 0.001). A statistically significant positive correlation was found between the subscale scores of microplastic pollution awareness and environmental literacy (*p* < 0.001).

**FIGURE 2:**
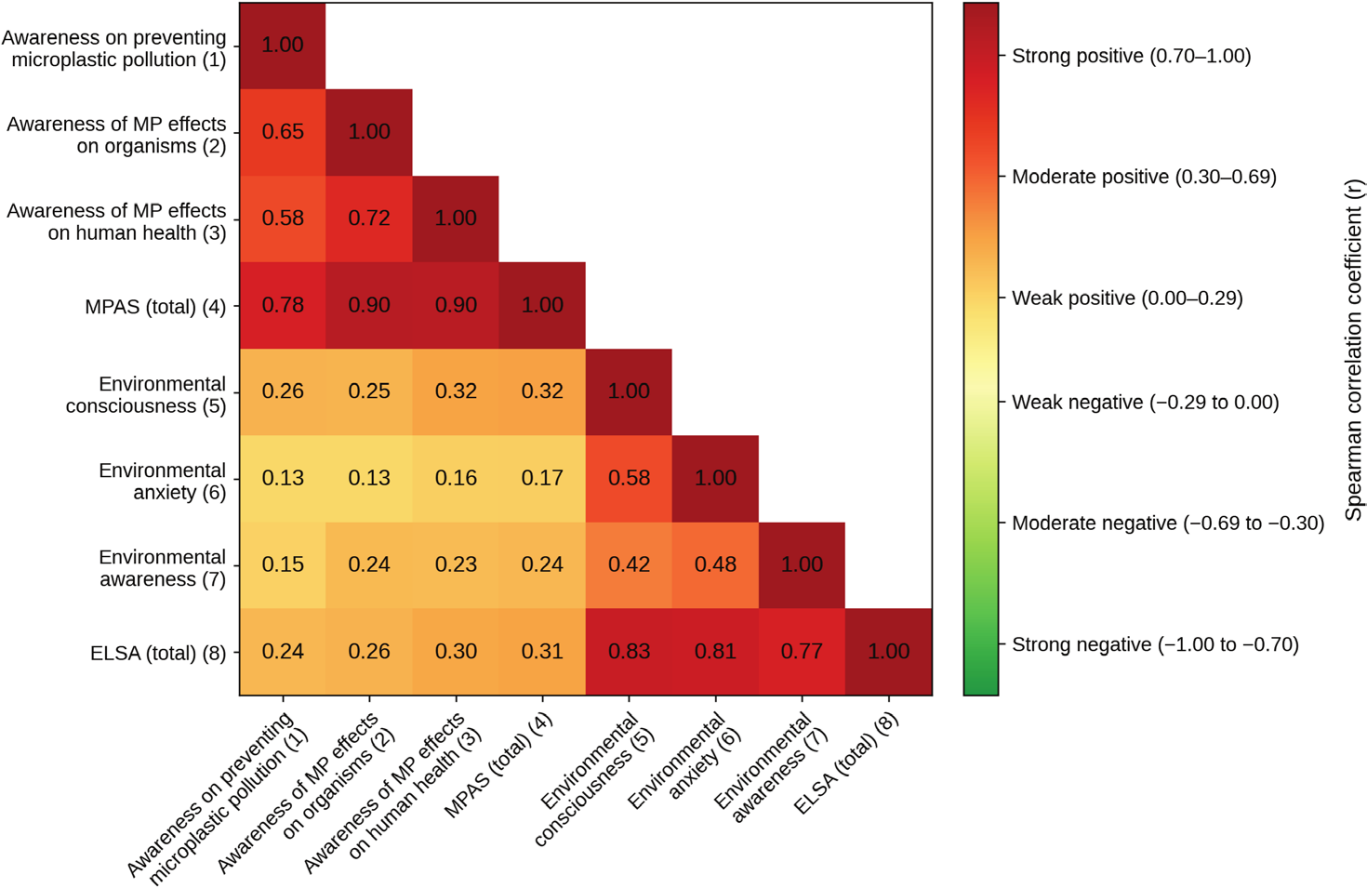
Correlation analysis between the study measures (*N* = 356).

The strongest relationship was found between awareness of the effects of microplastic pollution on human health and environmental consciousness (*r* = 0.320, *p* < 0.001). In addition, a positive, moderately strong, and significant relationship was found between the total score for microplastic pollution awareness and the environmental consciousness sub‐dimension (*r* = 0.321, *p* < 0.001) (Figure 2).

## 4 Discussion

Awareness among the younger generation about plastic and microplastic pollution, which has reached an alarming level today and is a threatening factor for future generations, is crucial for ensuring a sustainable and healthy environment. Nurses should carefully assess the patient's environmental health rather than just addressing their physical, social, and mental aspects, and include these in their care plans (Moniz et al. 2020; Riegel et al. 2021). As nurses and future professionals, nursing students need to determine new strategies against increasing environmental problems and microplastic levels. This study aims to examine the relationship between microplastic pollution awareness and environmental literacy levels among undergraduate nursing students. This study revealed that nursing students had a mean microplastic pollution awareness score of 22.98 ± 4.85 and a high level of environmental literacy.

However, a significant portion of students reported not having heard of the concept of microplastics before. Furthermore, students who expressed willingness to receive environmental education and those who had previously heard of microplastics demonstrated higher mean scores for both microplastic pollution awareness and environmental literacy. A significant positive relationship was found between microplastic pollution awareness and environmental literacy.

According to the study results, nursing students had a mean microplastic pollution awareness score of 22.98 ± 4.85. In the literature, studies on plastic pollution awareness have been conducted with nursing students. These studies indicate that the majority of nursing students demonstrate a moderate level of awareness of plastic pollution, while their knowledge regarding the health impacts of plastic pollution remains limited (Ravi, Edwin, and Jaison 2024; Ravi, Edwin, Rasheed, et al. 2024). In addition, previous research has reported that only a minority of nursing students possess sufficient knowledge about plastic pollution (Ravi et al. 2025). A study conducted with biology teachers observed that the mean scores of microplastic pollution awareness were similar to our study (Karakaya and Karakara 2023). In a study conducted with medical students, the mean microplastic pollution awareness score was 22.94 ± 3.67 (Uzun and Orhan 2025). A comparable mean score was observed in the present study, suggesting similar levels of awareness across these populations. This similarity may be related to shared educational backgrounds among health‐related students. In addition, nursing students' willingness to receive environmental education may contribute to their engagement with environmental issues and support the observed level of awareness. Awareness of microplastic pollution is important because perceived impacts, knowledge, and awareness of plastic and microplastic pollution influence pro‐environmental attitudes (Soares et al. 2021). Dowarah et al. (2022) found that participants with higher awareness of microplastic pollution were more willing to adopt behaviours to reduce plastic/microplastic pollution. Therefore, more studies are needed to measure nursing students' awareness of microplastic pollution. Despite the observed mean awareness score, our study results show that a significant majority of the students participating in this study, 68.3%, reported that they had not heard of the concept of microplastics before. This may be explained by the fact that, although participants are generally aware of the environmental impacts of plastics, they may lack structured training on the specific concept and terminology of microplastics. In contrast, studies from Germany, India and Türkiye reported that most university students were familiar with the term and could define it (Dowarah et al. 2022; Raab and Bogner 2021; Uzun and Orhan 2025). The lower level of familiarity with the concept of microplastics observed in the present study may be related to differences in educational curricula and exposure to environmental health content. According to our study's results, students who had previously heard of the concept of microplastics showed higher mean scores in environmental literacy and microplastic pollution awareness. This supports the finding that the increase in individuals' knowledge about microplastics is associated with pro‐environmental behaviours (Garcia‐Vazquez and Garcia‐Ael 2021). Therefore, to increase the awareness of microplastic pollution, it will be necessary to teach this concept to nursing students. Considering the increasing microplastic pollution, the concept of microplastics and their effects on human health should be included in the curriculum.

According to our study results, nursing students had a high level of environmental literacy. This is similar to other study findings in the literature (Amin et al. 2024; Cengiz and Bahar 2023; Dağal and Fırat Kılıç 2023; Incesu and Yas 2024; Yusuf et al. 2022). However, there are also studies showing that nursing students have moderate environmental literacy (Gök and Kiliç 2021; Kapan and Gürel 2022; Örs 2022). It was thought that the reason for these differences may be that environmental content is handled differently in the curricula and there is no standard. The environment and environmental content that directly affect human life should be included in all areas of the nursing curriculum, and students should be made aware of them through various practices. Furthermore, this study found that participants who were members of environmental organizations, who expressed a desire for environmental education, and who were familiar with the term ‘microplastics’ had significantly higher environmental literacy scores. This finding is in line with studies emphasizing the relationship between environmental awareness, environmental responsibility, and environmental literacy (Liu and Tobias 2024).

As a result of the study, a significant positive relationship was found between microplastic pollution awareness and environmental literacy. Although there are studies showing the relationship between environmental literacy and climate change awareness (Incesu and Yas 2024), environmental sensitivity (Yusuf et al. 2022), climate anxiety (Amin et al. 2024), and green consumption (Liu and Tobias 2024), no study has yet been found to examine the relationship with microplastic pollution. The environmental consciousness sub‐dimension showed the strongest relationship with microplastic pollution awareness and its sub‐dimensions. This finding suggests that individuals who perceive environmental issues as personally and socially significant are more likely to recognize and understand emerging environmental health threats such as microplastic pollution. This interpretation is consistent with evidence indicating that initiatives aimed at increasing environmental consciousness can effectively enhance microplastic pollution awareness (Bayoğlu and Uncu 2024). Environmental awareness and environmental education can also be an important strategy to reduce the consumption of plastics (Soares et al. 2021). Based on these findings, we interpret environmental literacy not merely as a knowledge‐based construct but as a multidimensional capacity that supports awareness, concern, and responsible action. Therefore, improving environmental literacy among nursing students represents an important step in the fight against microplastic pollution.

### 4.1 Strengths and Limitations

A key strength of this study is that it explores, for the first time, the relationship between microplastic pollution awareness and environmental literacy among undergraduate nursing students. It also assesses microplastic pollution awareness and the levels of environmental literacy in this group and identifies the factors influencing these variables—providing valuable insights that can inform improvements to the undergraduate nursing curriculum.

This study has several limitations. First, the data were collected from a single institution, which may limit the generalizability of the findings. Second, all data were based on self‐reported measures. Third, participants were recruited on a voluntary basis within proportionally stratified academic groups, indicating a convenience sampling approach. In addition, because the online survey was distributed via class communication groups, it was not possible to determine how many students received or viewed the survey link. Therefore, a precise response rate could not be calculated for the online component, which may have introduced selection bias. In addition, potential differences between online and face‐to‐face data collection methods were not assessed. Finally, one of the subscales of the Environmental Literacy Scale demonstrated borderline internal consistency (Cronbach's α = 0.692).

### 4.2 Nursing Implications

The findings of the study indicate a significant relationship between microplastic pollution awareness and environmental literacy. Based on this finding, strengthening environmental literacy among nursing students may be an effective strategy to raise awareness of microplastic pollution as an emerging public health issue. Given that microplastic pollution continues to increase and poses risks to ecosystems and human health, particularly to marine life, nursing education needs to address microplastics in the context of environmental and public health.

Notably, the finding that a substantial proportion of students had not previously heard of microplastics highlights a critical educational gap. This suggests a need for the systematic integration of microplastic‐related content into undergraduate nursing curricula and in‐service training programs.

In addition, the study found that students who participated in environmental organizations and those who expressed willingness to receive environmental education demonstrated higher mean scores in microplastic pollution awareness and environmental literacy. Therefore, nursing education programs should extend beyond theoretical instruction and actively promote participation in environmental initiatives, awareness campaigns, and practice‐based learning opportunities. Such approaches may support the development of sustained environmentally responsible behaviours among future nurses.

## 5 Conclusion

This study demonstrates that nursing students demonstrated microplastic pollution awareness alongside high levels of environmental literacy, with a positive and statistically significant association between these variables. The findings suggest that environmental literacy may play an essential role in shaping the understanding of emerging environmental health threats such as microplastic pollution.

However, because this study was conducted using a descriptive and correlational design and relied on self‐report data, causal relationships cannot be inferred. Additionally, as the study was conducted at a single institution, the findings cannot be generalized to all nursing students.

Future research should prioritize intervention‐based longitudinal studies to examine whether improvements in environmental literacy among nursing students and professionals translate into increased awareness of microplastic pollution, responsible plastic consumption, sustainable pro‐environmental behaviours, and sustainable lifestyle practices.

Complementary to this, there is a need for studies examining attitudes toward pro‐environmental behaviours and sustainable lifestyle practices, including factors associated with higher levels of microplastic pollution awareness among nurses and nursing students. Furthermore, qualitative studies are needed to identify the motivations and broader contextual factors that influence environmentally friendly behaviours.

## Author Contributions

Fatma Ceylan Çiray: selection problem, methodology, seeking ethical approval, writing – original draft and editing, statistical analysis, writing – original draft and editing. Ayşe Altınkaynak: data collection, collecting participants' data, writing and editing an original draft. Fatma Türedi and Tülay Erciyes: data collection, collecting participants' data, writing and editing an original draft. All authors agreed on the final version.

## Funding

This study was supported by Türkiye Bilimsel ve Teknolojik Araştırma Kurumu (TÜBİTAK), 2209‐A ‐ Research Project Support Programme for Undergraudate Students (2022/2 term), Grant numbers: 1919B012217718.

## Disclosure

Data analyses: The author, Fatma Ceylan Çiray, has basic statistics training. She also teaches research and epidemiology.

## Ethics Statement

The study received approval from the Research Ethics Commission at Hacettepe University (E‐35853172‐300‐00002516592), on 17 December 2022. In addition, institutional permission was obtained from the Hacettepe of Nursing Faculty, and permission was obtained from the scale owners for the scales to be used in the study. The research adhered to the ethical guidelines of the Helsinki Declaration. All participants were fully informed about the study's objectives and procedures and provided written informed consent prior to participation. Special attention was given to protecting vulnerable participants, particularly those who might feel coerced into participating due to the power dynamics inherent in an academic setting.

## Conflicts of Interest

The authors declare no conflicts of interest.

## Data Availability

The datasets generated and/or analyzed during the current study are available from the corresponding author on reasonable request.
